# Directivity Modeling and Simulation Analysis of a Novel Structure MEMS Piezoelectric Vector Hydrophone

**DOI:** 10.3390/mi14081495

**Published:** 2023-07-26

**Authors:** Wei Deng, Qingqing Fan, Junhong Li, Chenghao Wang

**Affiliations:** 1 State Key Laboratory of Acoustics, Institute of Acoustics, Chinese Academy of Sciences, Beijing 100190, China; 2 University of Chinese Academy of Sciences, Beijing 100049, China

**Keywords:** dual mass, directional offset, concave point depth, directional offset angle

## Abstract

In this paper, a novel dual-mass MEMS piezoelectric vector hydrophone is proposed to eliminate the transverse effect and solve the problem of directivity offset in traditional single-mass MEMS piezoelectric vector hydrophones. The reason for the directional offset of the traditional single-mass cantilever MEMS piezoelectric vector hydrophone is explained theoretically for the first time, and the angle of the directional offset is predicted successfully. Both analytical and finite element methods are employed to analyze the single-mass and dual-mass cantilever MEMS piezoelectric vector hydrophone. The results show that the directivity of the dual-mass MEMS piezoelectric vector hydrophone has no deviation, the transverse effect is basically eliminated, and the directivity (maximum concave point depth) is significantly improved, so more accurate positioning can be obtained.

## 1. Introduction

Almost 71% of the Earth’s surface is covered by water. In the future, humankind will rely heavily on marine resources. Therefore, high-performance underwater acoustic detection equipment, in which hydrophones are considered to be the core component [1], is urgently needed. Hydrophones mainly include acoustic pressure hydrophones and vector hydrophones. Compared with acoustic pressure hydrophones, vector hydrophones can measure vector information such as particle displacement, velocity, and acceleration of the underwater acoustic field. Combining MEMS technology with vector hydrophones is advantageous because this combination has a small size, low power consumption, low fabrication cost, and better signal noise ratio, and it can more easily detect low frequencies [2,3,4].

According to the sensing mechanism, MEMS vector hydrophones can be mainly divided into capacitive vector hydrophones [5,6], piezoresistive vector hydrophones [7,8,9,10,11,12], and piezoelectric vector hydrophones [13,14,15,16,17]. There are few articles available on capacitive MEMS vector hydrophones. Li et al. fabricated a low-noise capacitive MEMS vector hydrophone by employing differential capacitors with a sensitivity of −179.9 dB [5]. Among piezoresistive MEMS vector hydrophones, the bionic ciliated piezoresistive MEMS vector hydrophone stands out as a representative design. In 2007, Shang, Chen et al. proposed a bionic ciliated MEMS piezoresistive vector hydrophone with an unamplified sensitivity of −247.7 dB [9]. Building upon this design, they subsequently proposed variations, including the bionic T-Shape [8], bionic cup-shaped [10], and bionic fitness-wheel-shaped [12] MEMS piezoresistive vector hydrophones. Recently, a new design, the Crossed-circle MEMS ciliary vector hydrophone, has been proposed [11]. The article on the cup-shaped MEMS vector hydrophone reports the signal amplification factor with an unamplified sensitivity of −228.5 dB [10]. The latest Crossed-circle MEMS ciliary vector hydrophone does not specify the amplification factor while demonstrating an amplified sensitivity of −186.7 dB. In the realm of piezoelectric MEMS vector hydrophones, a significant contribution was made by Junhong Li et al. in 2016. They developed a cantilever MEMS vector hydrophone with ZnO thin films, which achieved an unamplified sensitivity of −229.5 dB [15]. In 2019, Dongning Li et al. proposed a novel MEMS vector hydrophone based on a composite beam with double U grooves, aiming to enhance sensitivity. This design achieved an unamplified sensitivity of −220.8 dB [4,18]. Capacitive mems vector hydrophones offer high sensitivity, but they need bias voltage during operation, are susceptible to the effects of parasitic capacitance, and possess complex structures. In contrast, piezoresistive and piezoelectric MEMS vector hydrophones have relatively low sensitivity, but they are passive devices with a simple structure. Furthermore, with continuous advancements, the sensitivity of piezoresistive and piezoelectric hydrophones has significantly improved. In comparison to piezoelectric MEMS vector hydrophones, the piezoresistive type is affected by unavoidable thermal noise caused by Joule heating. Additionally, its sensitivity is slightly lower than that of the piezoelectric type due to lower energy conversion efficiency [14,19,20].

However, the cantilever piezoelectric MEMS vector hydrophone exhibits a significant directional offset (transverse effect), leading to substantial errors in sound source localization [14,21,22]. The directional offset or transverse effect is manifested by the fact that the maximum sensitivity axis of the vector hydrophone does not coincide with the main axis [23]. For an excellent accelerometer, its transverse sensitivity ratio should be controlled within 5% or lower [21]. In 2002, K. Deng et al. introduced a ring-shaped piezoelectric MEMS accelerometer with a transverse sensitivity ratio of less than 2% and an acceleration of 0.77 pC/g [24]. In 2006, Lijie Chen et al. fabricated a piezoresistive MEMS vector hydrophone with bridge structure in order to reduce the large transverse effect of the cantilever structure. However, the transverse effect was not completely eliminated, and its directivity was still offset by approximately 5° [25,26]. In 2011, Chengzhe Li et al. proposed two compensation methods to reduce transverse effects based on the cymbal piezoelectric accelerometer, but both of them require multiple sensors to cooperate [27]. In 2014, Yan Liu et al. introduced an improved figure of merit (FOM) that encompasses resonant frequency, sensitivity, and transverse sensitivity [28]. In 2019, Lin lina et al. proposed a piezoresistive accelerometer with the non-planer dual flexure beam to reduce the influence of transverse effects. The transverse sensitivity ratio of this accelerometer was less than 0.078%, while the acceleration sensitivity was 0.64 mV/g [29]. In 2019, Jian Yang et al. proposed a T-Shape Piezoelectric MEMS Resonant Accelerometer with a transverse sensitivity ratio of less than 4.77% and an acceleration sensitivity of 1.11 Hz/g [30]. In the same year, to enhance the quality factor Q and sensitivity, Jian Yang et al. introduced a Piezoelectric MEMS Resonant Accelerometer with a fork-like structure and two proof masses resonate reversely, resulting in an acceleration sensitivity of 8.53 Hz/g and a transverse sensitivity ratio less than 6.1% [31]. In 2022, Chengying Li et al. proposed a square MEMS piezoelectric accelerometer with low transverse sensitivity. This accelerometer exhibited a sensitivity of 1.96 mV/g, and its transverse sensitivity ratio was less than 0.6% [21].

The transverse sensitivity of multi-beam structure can be well controlled, but the sensitivity is small. The sensitivity of the single-beam structure is large, but the transverse sensitivity is not well-controlled [25]. Currently, it is challenging to achieve both a low transverse sensitivity ratio and high sensitivity simultaneously. Cantilever MEMS piezoelectric vector hydrophones offer high sensitivity, but there is an urgent need to address the issue of directional offset (transverse sensitivity) without compromising their sensitivity. This paper aims to explore the underlying sensing mechanism causing directional offset and predict the angle of the directional offset in the cantilever MEMS piezoelectric vector hydrophone. Additionally, we propose a dual-mass MEMS piezoelectric vector hydrophone that addresses the issue of directional offset without compromising the high sensitivity of the cantilever structure.

## 2. Modeling Analysis

Vector hydrophones are primarily categorized into a moving-coil type, sound-pressure gradient type, and resonant-column type according to the principle of detecting vibrations. Different types of vibration detection principles correspond to different working principles, but they all share a common characteristic. That is, the detection of acoustic signals is directional, and the directional curve presents a pattern of “8” shape [32]. However, the directivity of the traditional single-mass MEMS piezoelectric vector hydrophone is offset, where the maximum concave point does not appear in the expected 90° and 270° [14,15]. In this section, the reasons for the directional offset of the traditional single-mass MEMS piezoelectric vector hydrophone will be explored, and the corresponding solutions will be proposed.

The directivity of a vector hydrophone is defined as a function of the output voltage [33]:

(1) $$D(\theta )=20\log(\frac{\left|V(\theta )\right|}{\left|V(\theta_{0})\right|})$$

where |V(θ)| is the absolute value of the output voltage of the vector hydrophone when the incident acoustic wave is along with the incident angle of θ, and $\left|V(\theta_{0})\right|$ is the absolute value of the maximum output voltage of the vector hydrophone when the incident acoustic wave is along with the incident angle of $\theta_{0}$. Equation (1) shows that the maximum concave point occurs at the minimum output voltage.

To simplify the analysis of the MEMS vector hydrophone, the following assumptions are made: 1. The effective mass of the cantilever beam is significantly smaller than that of the proof mass and can be neglected; 2. The layers of the cantilever beam are elastic and obey Hooke’s law; 3. The proof mass and the cantilever beam are rigidly connected; 4. The cantilever beam undergoes pure bending deformation, while the stresses in the z direction and the strains in the x direction can be ignored compared with the other strains and stresses [34].

When the traditional single-mass MEMS piezoelectric vector hydrophone is subjected to acceleration in the y-z plane, it can be simplified to the following model.

**Figure 1:**
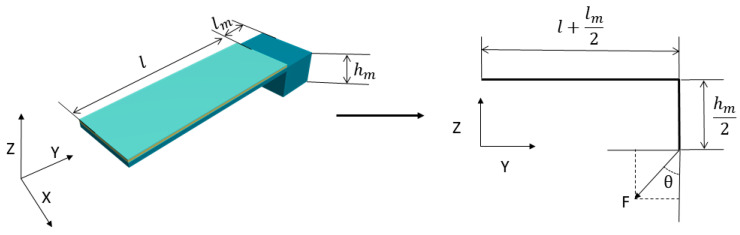
Simplified model of the traditional single-mass MEMS piezoelectric vector hydrophone.

In Figure 1, $l_{m}$ and $h_{m}$ are the length and height of the proof mass, and l is the length of the cantilever beam. When the single-mass MEMS piezoelectric vector hydrophone is subjected to an acceleration $a_{cc}$, then $F=ma_{cc}$ equates the force point to the center of the proof mass.

The orthogonal decomposition of F is obtained as follows:

(2) $$F_{a}=F\cos\theta F_{b}=F\sin\theta$$

The bending moment generated by $F_{b}$ at the vertex of the point cantilever beam is

(3) $$M_{b}=F_{b}\frac{h_{m}}{2}$$

The bending moment at any position on the composite beam is

(4) $$M(x)=M_{b}+F_{a}(l+\frac{l_{m}}{2}-x)=F\sin\theta \frac{h_{m}}{2}+F\cos\theta (l+\frac{l_{m}}{2}-x)$$

The average stress of the piezoelectric film is

(5) $$\sigma_{1}=E_{p}\frac{M(x)}{{\left(EI\right)}_{eq}}\left(\frac{h_{p}}{2}+a\right)$$

where $E_{p}$ is young modulus of the piezoelectric film, $h_{p}$ is the thickness of the piezoelectric film, a is the distance between the neutral surface of the composite beam and the piezoelectric layer, and ${\left(EI\right)}_{eq}$ is equivalent flexural rigidity of the composite beam [34].

The output voltage of the piezoelectric layer due to acceleration is

(6) $$V(\theta )=\frac{Q}{C}=\frac{1}{C}\int_{0}^{l_{e}}d_{31}\sigma_{1}bdx=\frac{bd_{31}E_{p}}{C{\left(EI\right)}_{eq}}\left(\frac{h_{p}}{2}+a\right)\int_{0}^{l_{e}}M(x)dx=A\int_{0}^{l_{e}}M(x)dx$$

where $l_{e}$ is the length of the electrode and $d_{31}$ is the piezoelectric coefficient of the piezoelectric film. For the determined composite beam, its related parameter A is also determined and can be regarded as a constant. According to Equations (4) and (6), the output voltage generated by the traditional single-mass MEMS piezoelectric vector hydrophone is the result of the combined action of the vertical and horizontal components of the acceleration.

According to Equation (6), the charge quantity of the piezoelectric element is the integral of the bending moment. When the bending moment of the traditional single-mass MEMS piezoelectric vector hydrophone changes sign in the electrode, the tensile stress on one end of the piezoelectric film and the compressive stress on the other end will generate charges of opposite polarity, which cancel each other out, resulting in the deviation of directivity of the traditional single-mass MEMS piezoelectric vector hydrophone.

Setting V(θ)=0 solves the concave point position θ in the y–z plane of the traditional single-mass MEMS piezoelectric vector hydrophone.

(7) $$\theta =\arctan(\frac{-2l-l_{m}+l_{e})}{h_{m}})$$

where l is the length of the composite beam, $l_{e}$ is the length of the electrode, and $l_{m}$ and $h_{m}$ are the length and height of the proof mass.

**Figure 2:**
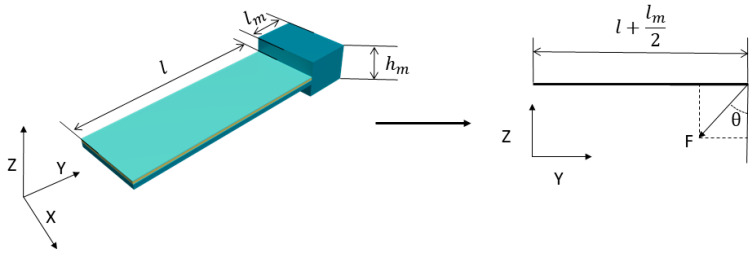
Simplified model of the dual-mass MEMS piezoelectric vector hydrophone.

To correct the directional offset of the traditional single-mass MEMS piezoelectric vector hydrophone, a dual-mass MEMS piezoelectric vector hydrophone with an upper and lower symmetry is proposed. The simplified model of the dual-mass MEMS piezoelectric vector hydrophone is shown in Figure 2.

The bending moment and output voltage of the dual-mass MEMS piezoelectric vector hydrophone are

(8) $$M_{b}=F_{b}\times 0=0$$

(9) $$M=M_{b}+F_{a}(l-x)=F\cos\theta (l-x)$$

(10) $$V(\theta )=\frac{Q}{C}=\frac{1}{C}\int_{0}^{l_{e}}D_{3}bdx=A\int_{0}^{l_{e}}F\cos\theta (l-x)dx$$

where $l_{e}$ is the length of the electrode. According to Equation (10), when θ is 90° and 270°, V(θ)=0. The concave point appeared at 90° and 270°, and the directivity did not deviate. The proof masses of the traditional single-mass and dual-mass MEMS piezoelectric vector hydrophone have the same weight. However, the dual-mass MEMS piezoelectric vector hydrophone makes the center of gravity of the proof mass located in the plane of the composite beam through the symmetric proof masses, thus solving the directional offset of the traditional single-mass MEMS vector hydrophone.

Based on Equations (6) and (10), the charge generated by the single-mass MEMS piezoelectric vector hydrophone is influenced by both the horizontal and vertical components of acceleration. In contrast, the dual-mass MEMS piezoelectric vector hydrophone is only sensitive to the vertical component of acceleration. Therefore, more accurate sound source location can be obtained.

## 3. Finite Element Analysis

Some approximate assumptions have been made in the above analytical analysis. In this section, finite element simulation is used to analyze the directivity of the MEMS piezoelectric vector hydrophone.

**Figure 3:**
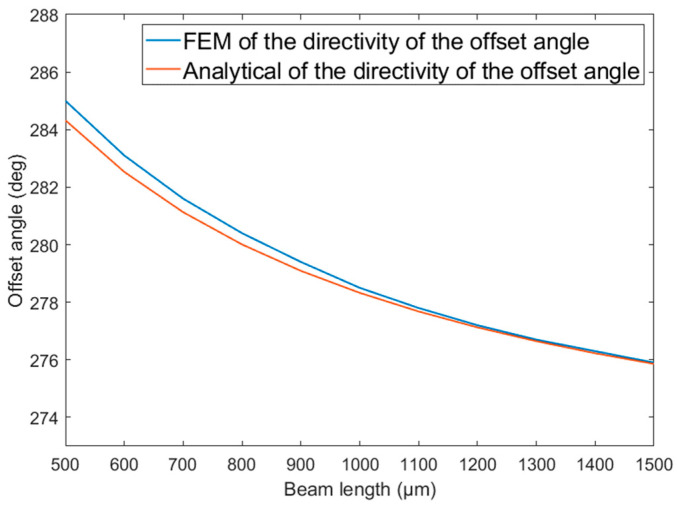
Directional offset angle of the traditional single-mass MEMS piezoelectric vector hydrophone as a function of the beam length. $l_{m}=300\;\mu m,\;b=300\;\mu m,\;h_{p}=4\;\mu m,\;h=8\;\mu m,\;l_{e}=\frac{l}{2}\mu m,\;h_{m}=300\;\mu m$.

Figure 3 shows the relationship between the directional offset angle of the traditional single-mass MEMS piezoelectric vector hydrophone and the length l of the composite beam. As shown in Figure 3, the directional offset angle decreases gradually as the length l of the composite beam increases.

**Figure 4:**
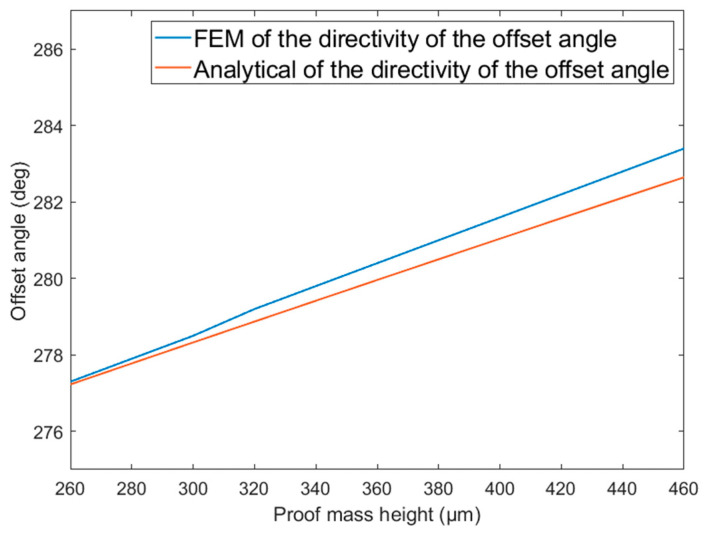
Directional offset angle of the traditional single-mass MEMS piezoelectric vector hydrophone as a function of the proof mass height. ($l_{m}=300\;\mu m,\;b=300\;\mu m,\;h_{p}=4\;\mu m,\;h=8\;\mu m,\;l_{e}=500\;\mu m,\;l=1000\;\mu m$).

**Figure 5:**
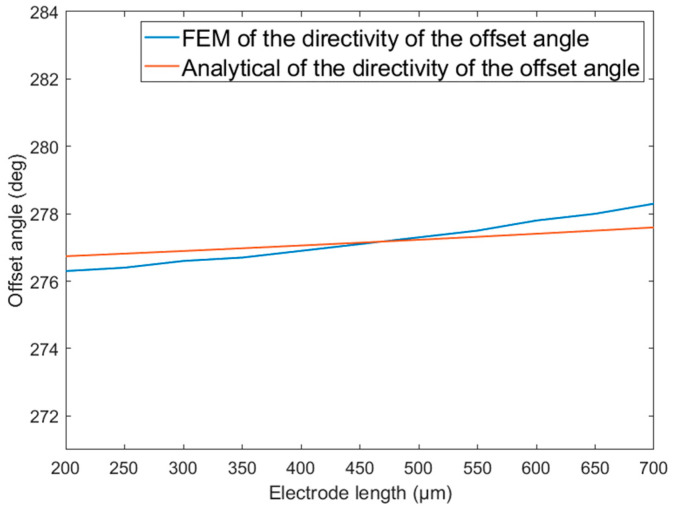
Directional offset angle of the traditional single-mass MEMS piezoelectric vector hydrophone as a function of the electrode length. ($l_{m}=300\;\mu m,\;b=300\;\mu m,\;h_{p}=4\;\mu m,\;h=8\;\mu m,\;h_{m}=260\;\mu m,\;l=1000\;\mu m$).

Figure 4 and Figure 5 show the variation in the directional offset angle of the traditional single-mass MEMS piezoelectric vector hydrophone with proof mass height $h_{m}$ and electrode length $l_{e}$, respectively. As shown in Figure 4 and Figure 5, with increasing proof mass height $h_{m}$ or electrode length $l_{e}$, the directional offset angle of the traditional single-mass MEMS piezoelectric vector hydrophone increases. The electrode length $l_{e}$ has little effect on the directional offset angle.

As observed from Figure 3, Figure 4 and Figure 5, regarding the directional offset angle of the traditional single-mass MEMS piezoelectric vector hydrophone, the finite element simulation results are basically consistent with the theoretical modeling results in the above section, and the maximum error is less than 0.8°.

**Figure 6:**
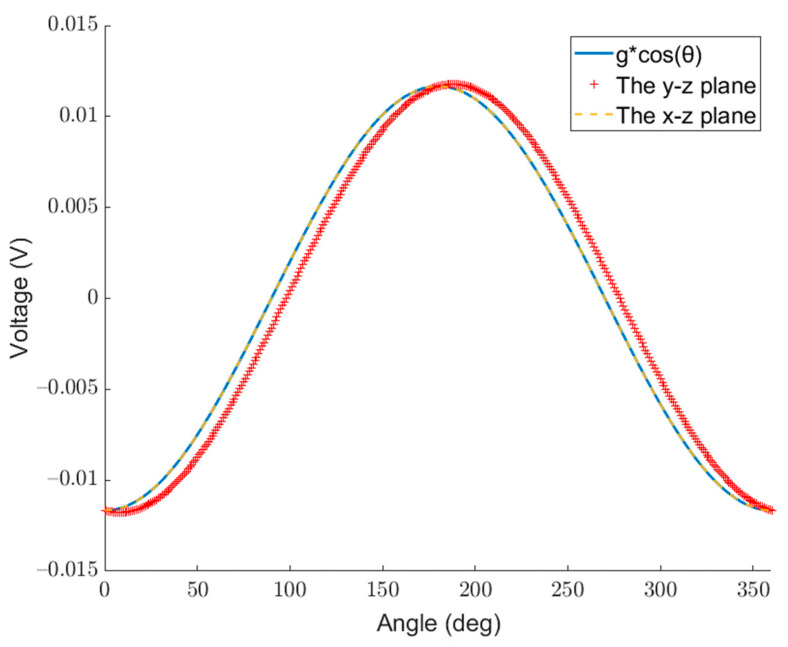
Voltage generated by the acceleration of different magnitudes in different directions for the traditional single-mass MEMS piezoelectric vector hydrophone. $(l_{m}=300\;\mu m,\;\;b=300\;\mu m,\;h_{p}=4\;\mu m,\;h=8\;\mu m,\;l_{e}=500\;\mu m,\;l=1000\;\mu m,\;h_{m}=280\;\mu m$).

**Figure 7:**
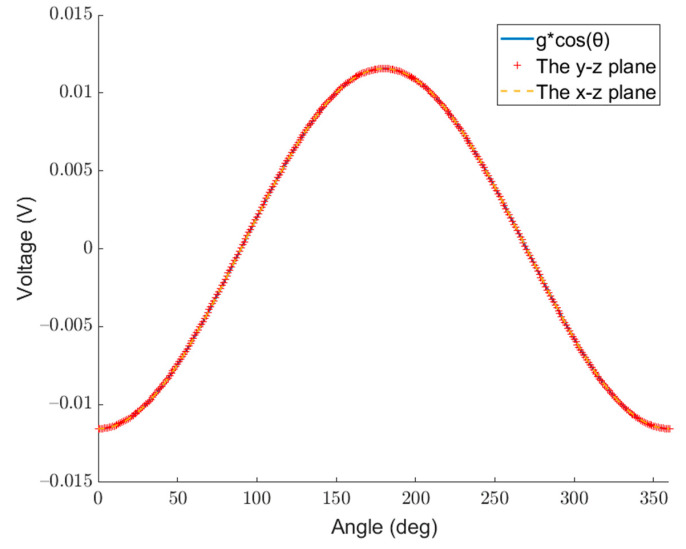
Voltage generated by the acceleration of different magnitudes in different directions for the dual-mass MEMS piezoelectric vector hydrophone. ($l_{m}=300\;\mu m,\;b=300\;\mu m,\;h_{p}=4\;\mu m,\;h=8\;\mu m,\;l_{e}=500\;\mu m,\;l=1000\;\mu m,\;h_{m}=140+140\;\mu m$).

Figure 6 and Figure 7 show the voltages generated by the acceleration of different magnitudes in different directions for the traditional single-mass and the dual-mass MEMS piezoelectric vector hydrophone, respectively. The curve with the dotted line and “+” sign represents the voltage generated by the single-mass MEMS piezoelectric vector hydrophone and the dual-mass one with constant acceleration at different angles in the y–z plane and the x–z plane. The solid line represents the voltage generated by different magnitudes of acceleration (g∗cos⁡(θ)) in a fixed direction (z direction).

As shown in Figure 6, the curve of g∗cos⁡(θ) in the fixed direction of the traditional single-mass MEMS piezoelectric vector hydrophone coincides with the curve of constant acceleration in the x–z plane at different angles. This indicates that the effect produced by acceleration at different angles in the x–z plane can be completely equivalent to the effect produced by g∗cos⁡(θ) acceleration along the z direction. g∗cos⁡(θ) can be considered as the vertical component of the constant acceleration at different angles.

However, the curve of g∗cos⁡(θ) in the fixed direction of the traditional single-mass MEMS piezoelectric vector hydrophone does not coincide with the curve of constant acceleration in the y–z plane at different angles. This indicates that the effect produced by acceleration at different angles in the y–z plane cannot be equivalent to the effect produced by g∗cos⁡(θ) acceleration along the z direction. This is consistent with the analysis in the previous section. The charge generated by the traditional single-mass MEMS piezoelectric vector hydrophone in the y–z plane is the result of the combined action of the horizontal component and the vertical component of acceleration, which leads to directional offset.

Figure 7 shows that for the dual-mass MEMS piezoelectric vector hydrophone, the curve of g∗cos⁡(θ) in the fixed direction coincides with the curve of the x–z plane and the curve of the y–z plane. This indicates that when it is subjected to an acceleration in any direction, the acceleration can be decomposed orthogonally. The voltage generated by the dual-mass MEMS piezoelectric vector hydrophone is equivalent to the result of the independent action of the acceleration component perpendicular to the dual-mass MEMS piezoelectric vector hydrophone. The dual-mass MEMS piezoelectric vector hydrophone is only sensitive to the vertical component of acceleration, and the voltage generated is only the result of the vertical component acting alone. Compared with the traditional single-mass MEMS piezoelectric vector hydrophone, the dual-mass MEMS piezoelectric vector hydrophone is more accurate in terms of sound source localization.

**Figure 8:**
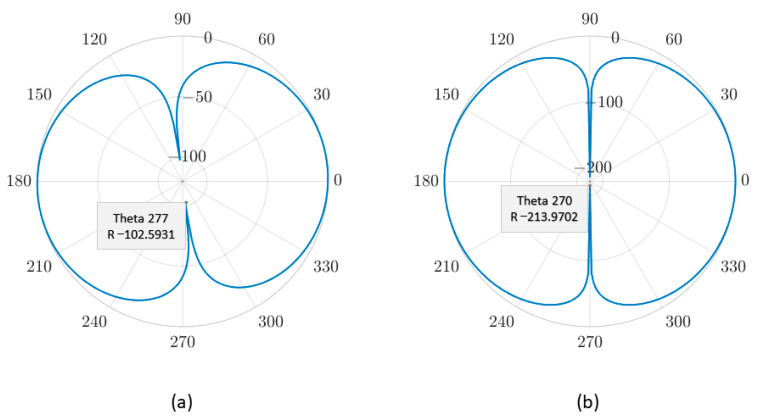
Directivity of the traditional single-mass MEMS piezoelectric vector hydrophone. (**a**) The y–z plane. (**b**) The x–z plane. ($l_{m}=300\;\mu m,\;b=300\;\mu m,\;h_{p}=4\;\mu m,\;h=8\mu m,\;l_{e}=500\;\mu m,\;l=1000\;\mu m,\;h_{m}=260\;\mu m$).

**Figure 9:**
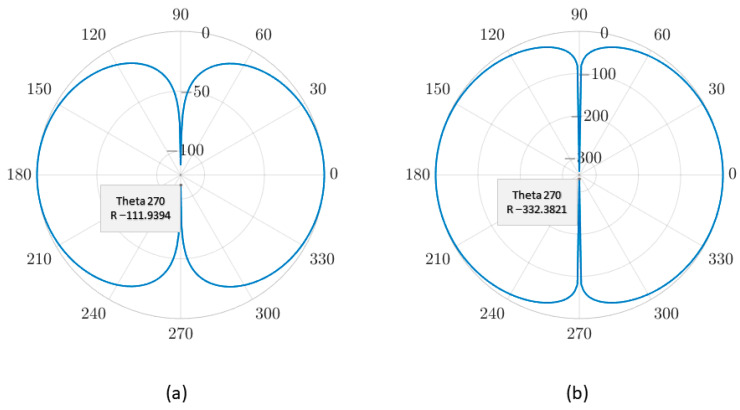
Directivity of the dual-mass MEMS piezoelectric vector hydrophone. (**a**) The y–z plane. (**b**) The x–z plane. ($l_{m}=300\;\mu m,\;b=300\;\mu m,\;h_{p}=4\;\mu m,\;h=8\;\mu m,\;l_{e}=500\;\mu m,\;l=1000\;\mu m,\;h_{m}=130+130\;\mu m$).

The directivity of the traditional single-mass and the dual-mass MEMS piezoelectric vector hydrophone are shown in Figure 8 and Figure 9, respectively. The −z direction is the starting 0°.

Figure 8 and Figure 9 show that the single-mass and the dual-mass MEMS piezoelectric vector hydrophone exhibit clear directivity patterns of “8” shape. However, in the y–z plane, the “8” shape directivity of the traditional single-mass MEMS piezoelectric vector hydrophone is offset, and its concave point appears at 277° instead of 270°. The concave point of the dual-mass MEMS piezoelectric vector hydrophone appears at 270°, and the directivity pattern of “8” shape is not offset.

In the y–z plane, the maximum concave point depths of the single-mass and the dual-mass MEMS piezoelectric vector hydrophone are −102.59 dB and −111.94 dB, respectively. In the x–z plane, the maximum concave point depths of the traditional single-mass and the dual-mass MEMS piezoelectric vector hydrophone are −213.97 dB and −332.38 dB, respectively. Compared with the traditional single-mass MEMS piezoelectric vector hydrophone, the maximum concave point depth of the dual-mass MEMS piezoelectric vector hydrophone is increased by 118.42 dB. The directivity performance is significantly improved.

The transverse sensitivity ratio refers to the ratio of transverse sensitivity to the sensitivity along the main sensitive axis [22,35]. In this study, it is represented as $\frac{S(90{}^{\circ})}{S(0{}^{\circ})}$, where S(90°) and S(0°) indicate the sensitivity of the vector hydrophone at 90° and 0° acceleration directions, respectively. The transverse sensitivity ratio for the *y*-axis of the single-mass MEMS piezoelectric vector hydrophone is 12.88%, while for the dual-mass one, it is 0.37%. These results demonstrate that the proposed dual-mass MEMS piezoelectric vector hydrophone effectively eliminates the transverse effect.

**Figure 10:**
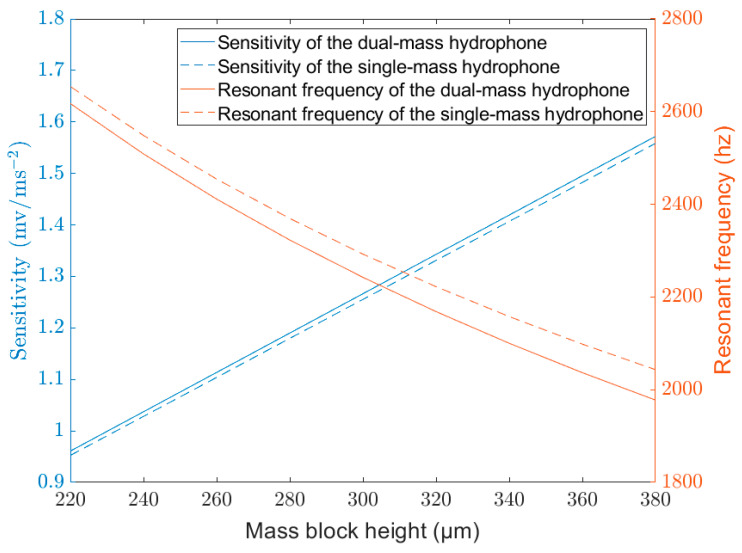
Resonant frequency and sensitivity of the single-mass and dual-mass MEMS piezoelectric vector hydrophone. ($l_{m}=300\;\mu m,\;b=300\;\mu m,\;h_{p}=4\;\mu m,\;h=8\;\mu m,\;l_{e}=500\;\mu m,\;l=1000\;\mu m$).

Table 1 shows the sensitivity and directional offset of various types of MEMS vector hydrophones. The data of the first five vector hydrophones in Table 1 are experimental data, and the data of this work are theoretical. The table indicates that other vector hydrophones exhibit directional offset issues, while the proposed dual-mass MEMS piezoelectric vector hydrophone addresses this problem. The resonant frequency (typically obtained through impedance measurement system) is another important metric for vector hydrophones, as it determines the bandwidth of the hydrophone [36]. Figure 10 shows the sensitivity and resonant frequency of the single-mass and dual-mass MEMS piezoelectric vector hydrophone as a function of the proof mass height. Here, the proof mass height of the dual-mass MEMS piezoelectric vector hydrophone is the sum of the upper and lower proof masses. It can be observed that the dual-mass hydrophone exhibits slightly higher sensitivity and slightly lower resonant frequency compared to the single-mass hydrophone. These characteristics are advantageous for detecting low-frequency signals.

## 4. Conclusions

In this paper, a comprehensive analysis of the directivity of the single-mass cantilever MEMS piezoelectric vector hydrophone is conducted for the first time. The results reveal that the traditional single-mass MEMS piezoelectric vector hydrophone is affected by both vertical and horizontal components of acceleration, which leads to directional offset. The analytical method and finite element simulation are used to analyze the directional offset angle of the traditional single-mass MEMS piezoelectric vector hydrophone. The results show that the analytical method aligns with the finite element simulation outcomes, validating the accuracy of the analytical method in predicting the directional offset angle.

To solve the directional offset issue of the traditional single-mass MEMS piezoelectric vector hydrophone, a novel dual-mass MEMS piezoelectric vector hydrophone is proposed in this paper. By employing symmetric proof masses, the center of gravity of the proof masses is positioned at the middle plane of the composite cantilever so that the dual-mass MEMS piezoelectric vector hydrophone is only sensitive to the vertical component of the acceleration. Therefore, the directional offset of the traditional single-mass MEMS piezoelectric vector hydrophone is corrected, and the transverse effect is basically eliminated. In comparison to single-mass MEMS vector hydrophones, the transverse sensitivity ratio of the dual-mass MEMS vector hydrophone is optimized from 12.88% to 0.37%, with slightly higher sensitivity and slightly lower resonant frequency. The voltage generated by the dual-mass MEMS piezoelectric vector hydrophone is only influenced by the sensitive direction and not affected by other directions. Therefore, a more accurate sound source location can be obtained. The maximum concave depth of the dual-mass MEMS piezoelectric vector hydrophone in the x–z plane and y–z plane is increased by 118.42 dB and 9.35dB, respectively, and the directivity performance is greatly improved. The directivity of the single-mass MEMS piezoelectric vector hydrophone is offset by 7° in the y–z plane, while the dual-mass MEMS piezoelectric vector hydrophone has no offset. However, it should be noted that the fabrication process of the dual-mass MEMS piezoelectric vector hydrophone is more complex and challenging compared to the single-mass one.

## Figures and Tables

**Table 1:** Sensitivity and directivity offset of various types of MEMS vector hydrophones.

Works	Sensing	Structure	Sensitivity	Directional Offset
Lijie Chen. et al. [25,26]	Piezoresistive	Bridge four cantilever	−194 dB (Amplification 53 dB)	Yes
Wei Xu. et al. [10]	Piezoresistive	Bionic cup-shaped	−188.5 dB (Amplification 40 dB)	Yes
Jinlong Song. et al. [19]	Piezoresistive	Bio-inspired	X-channel: −187 dB Z-channel: −163 dB (With amplification)	Yes
Junhong Li. et al. [15]	Piezoelectric	Cantilever	−229.5 dB (No amplification)	Yes
Qingqing Fan. et al. [4,18]	Piezoelectric	Cantilever with double U groove	−186.8 dB (Amplification 34 dB)	Yes
This work	Piezoelectric	Dual-mass Cantilever	slightly higher than cantilever [15]	No

## Data Availability

Not applicable.
